# Lasers in Transurethral Enucleation of the Prostate—Do We Really Need Them

**DOI:** 10.3390/jcm9051412

**Published:** 2020-05-10

**Authors:** Thomas R.W. Herrmann, Stavros Gravas, Jean JMCH de la Rosette, Mathias Wolters, Aristotelis G. Anastasiadis, Ioannis Giannakis

**Affiliations:** 1 Department of Urology, Spital Thurgau AG, Frauenfeld, 8569 Münsterlingen, Switzerland; aristotelis.anastasiadis@stgag.ch (A.G.A.); ioannis.giannakis@stgag.ch (I.G.); 2 Department of Urology, Hannover Medical School, 30625 Hannover, Germany; Wolters.Mathias@mh-hannover.de; 3 Department of Urology, University Hospital of Larisa, 41500 Larisa, Greece; sgravas2002@yahoo.com; 4 Department of Urology, Istanbul Medipol University, Istanbul 34214, Turkey; j.j.delarosette@gmail.com

**Keywords:** HoLEP, ThuLEP, ThuVEP, GreenLEP, Vapoenucleation, AEEP, EEP, laser, Holmium, Thulium, diode, Greenlight, LBO

## Abstract

The acronym EEP, coding for transurethral Endoscopic Enucleation of the Prostate, was introduced in 2016 by the European Association of Urology (EAU) guidelines panel on management of non-neurogenic male lower urinary tract symptoms (LUTS) and benign prostatic obstruction (BPO). Since then, a laser-based treatment, Holmium Laser Enucleation of the Prostate (HoLEP), and the current-based treatment of bipolar enucleation of the prostate (BipoLEP) are equally appreciated as valuable options for the management of benign prostatic obstruction (BPO). This was mainly inspired by the results of two meta-analyses on randomized controlled trials, comparing open prostatectomy with either Holmium Laser Enucleation of the Prostate (HoLEP) or bipolar enucleation of the prostate (BipoLEP). Prior to that, HoLEP was embraced as the only valid option for transurethral enucleation, although evidence for equivalence existed as early as 2006, but was not recognized due to a plethora of acronyms for bipolar energy-based treatments and practiced HoLEP-centrism. On the other hand, the academic discourse focused on different (other) laser approaches that came up, led by Thulium:Yttrium-Aluminum-Garnet (Tm:YAG) Vapoenucleation (ThuVEP) in 2009 and, finally, transurethral anatomical enucleation with Tm:YAG support (thulium laser enucleation of the prostate, ThuLEP) in 2010. Initially, the discourse on lasers focused on the different properties of lasers rather than technique or surgical anatomy, respectively. In and after 2016, the discussion ultimately moved towards surgical technique and accepting anatomical preparation as the common of all EEP techniques (AEEP). Since then, the unspoken question has been raised, whether lasers are still necessary to perform EEP in light of existing evidence, given the total cost of ownership (TCO) for these generators. This article weighs the current evidence and comes to the conclusion that no evidence of superiority of one modality over another exists with regard to any endpoint. Therefore, in the sense of critical importance, AEEP can be safely and effectively performed without laser technologies and without compromise.

## 1. Introduction

The evolution of Endoscopic Enucleation of the Prostate (EEP) began humbly in 1983, with the introduction of the blueprint of all transurethral anatomical enucleating techniques, using a prostate detachment probe to dissect along the false capsule of fibrous tissue between the peripheral zone and adenoma (Hiroaoka 1983) [1]. It was not until 2005 when Frauendorfer and Gilling developed and introduced the pulsed Holmium-Laser and, subsequently, the Holmium-Laser Enucleation of the Prostate (HoLEP) into clinical practice [2]. However, despite excellent clinical results in randomized controlled trials, HoLEP did not become widely embraced in the urological community. 

After the introduction of the Thulium:Yttrium-Aluminum-Garnet (YAG) Laser in 2005, the HoLEP establishment was challenged by the introduction of a number of transurethral techniques for the treatment of the prostate using new energy sources. The working groups, thereby, mainly replicated the path that former Holmium:YAG (Ho:YAG) promoters had taken by subsequently introducing vaporizing, incising, resecting and, finally, enucleating techniques. 

Thulium Vapoenucleation of the prostate (ThuVEP) data, focusing on the vapo-incising capacity of the novel Thulium laser, were published in 2009 by Bach et al. [3]. The template removed resembled the transurethral enucleation appearance, with the difference being that the incising and simultaneous sealing of the cutting plane did not match the visual expectations, compared to the appearance of a surgical surface after HoLEP. Inspired by the academic discussion (whether enucleation can only be performed by a Holmium laser) Herrmann et al. [4] proposed a concept of anatomical enucleation by a widely blunt dissection of the transitional zone (ThuLEP) in 2010. The Thulium laser, in this context, was used and proposed as an assisting tool for incision and coagulation only.

The scientific discussion on Thulium:YAG as the prototype for all other continuous wave lasers acted as an ignition for the further development of enucleating techniques. The surgical twins “ThuVEP” and “ThuLEP” demonstrated the whole spectrum of laser action in enucleation techniques. ThuVEP focused on the favorable vaporization effect of continuous wave lasers to perform a fast, effective, and safe enucleation, whereas ThuLEP focused on almost blunt mechanical enucleation using the laser only for dissection of adherences and incising of the mucosa. The principle of the latter, on an anatomy focusing approach, was mimicked by other continuous wave lasers (lithium triborate, LBO/“Greenlight” and 980 nm diode laser) [5,6], whereas before it had not been found suitable for enucleation [7,8,9].

In the beginning of the HoLEP era, transurethral plasmakinetic (i.e., bipolar) enucleation of the prostate (PkEP) was developed, in 2004, by the same group of HoLEP innovators. Although bipolar enucleation of the prostate had already demonstrated equivalence to HoLEP in a randomized controlled trial in 2006 [10], it took two meta-analyses in 2015 [11,12] to unveil the convincing evidence of enucleating techniques based on bipolar energy. These studies analyzed the results of randomized controlled trials comparing open prostatectomy with either Holmium Laser Enucleation of the Prostate (HoLEP) or bipolar enucleation of the prostate (BipoLEP). They concluded equivalence with regard to efficacy to relief obstruction, perioperative safety, and long-term results. Consequently, the 2016 update of the European Association of Urology (EAU) guidelines on management of non-neurogenic male lower urinary tract symptoms (LUTS), with the reception of two meta-analyses [13], promoted both HoLEP and bipolar enucleation as the treatment of choice for benign prostatic obstruction (BPO) of large volume prostatic glands. This resulted in the change of scientific reception—away from highlighting the impact of a single energy source (back) to the overarching principle of the common ground of enucleating techniques: endoscopic enucleation (EEP) or anatomical enucleation (AEEP).

Inspired by these novel techniques, a growing interest in the urological community could be observed. This interest was further enhanced by the introduction of novel enucleation probes and the presentation and discussion in many live surgical events. Furthermore, EEP carried out with high frequency (HF) current based energy sources potential allows dissemination of transurethral enucleating techniques in healthcare systems and socio-economic environments with restricted financial budgets, since the capital investment costs in energy delivery systems, such as laser generators, can be considered an obstacle (total cost of ownership, TCO). 

The academic discourse on AEEP today has moved toward measures to prevent early postoperative stress urinary incontinence and retrograde ejaculation. Furthermore, variations of the original technique were introduced, such as the 2-lobe approach [14,15] and the en bloc dissection [16]. 

The basic difference between the 3 lobe, 2 lobe [14,15,17], and en bloc enucleation [16] is the incision into the mucosa: ventrally (“double W/U” (Endoskils 2016), “three horseshoe” [18], and the incision of the mucosa to dissect the transitional zone in 3 lobes (3), two lobes (2), or the entire lobe/en bloc (0). 

The entire transitional zone enucleation “en bloc AEEP/EEP”, such as in open prostatectomy, has been popularized, mainly by third generation HoLEP promoters, and has gained momentum ever since. The overarching principle of all approaches using either Ho:YAG or Thulium (Tm):YAG lasers is anatomical preparation, anteroposterior dissection, early release of the ventral mucosa, and apical mucosa sparing incisions, in order to reduce early postoperative incontinence (and ease the learning curve by achieving several reproducible quality markers) (Pentafecta) [5,16,18,19,20,21,22].

Additionally, mechanical tissue morcellation as an integral part of AEEP and is often seen as an obstacle because it might be a potential source of additional complications, a prolonged learning curve, and additional capital investment costs.

In the last 5 years, the Thulium fiber laser has been gaining attention, although technically it has mainly been used in soft tissue since 2005 [23]. However, in the advent of using Thulium fiber laser as a versatile tool for kidney stones management (as a dusting tool), soft tissue effects have come into focus again. Thus far, the difference of wavelengths from 1940 to 2013 nm has not translated into different clinical outcomes [24,25]. The future main academic conflict demarcation lines will most likely be located in between Tm fiber lasers and high power high frequency Ho:YAG generators, with different pulse modulations [26].

The third wave of industry marketing has led to another round of debates on the role of laser energy sources by Ho:YAG laser pulse modulation (the so-called Moses Effect/Technique) [26] and novel thulium fiber laser generators [27], thereby mimicking the debate of the 2000s, but this time crab walking back to, at times, below the scientific level reached before. 

The academic consensus of today positions “concept” over “energy source”, i.e., the energy source has a secondary impact on the success of AEEP and seems to be, rather, a result of institutional resources, or personal preference of a skilled surgeon; transurethral endoscopic enucleations have the same given effect, they are anatomical EEP [8,28,29,30,31].

In the given context of a novel generation of laser generators, the question was raised if lasers, in general, are of essential impact in AEEP in light of a less costly but, nonetheless, equally effective option—bipolar enucleation. The present article aims to enlighten this daring thesis and to inspire a reflection on “essentiality” of energy sources for AEEP.

## 2. Body of Evidence

The body of literature dealing with lasers has exponentially increased in the past 10 years. However, randomized controlled data with low risk of bias, imprecision, inconsistency, indirectness, and publication bias are scarce. High quality randomized controlled trials comparing the different energy sources are even less commonly available. 

Currently, the 2020 update of the EAU guidelines panel on management of non-neurogenic male lower urinary tract symptoms (LUTS), including benign prostatic obstruction (BPO) [32], has summarized the data of the past years. In addition, the latest collaborative systemic review and cumulative analysis [33] of the EAU Section of Uro-Technology (ESUT) on the outcome of bipolar enucleation vs. transurethral resection of prostate (TURP) in 2020, and the two meta-analyses of bipolar enucleation vs. transvesical open prostatectomy of Lin et al. in 2015, represent a summary of the body of evidence for bipolar enucleation compared to the standard treatment arm, and are also inherently not qualified to answer to the above raised question (whether lasers are needed in the context of transurethral enucleation of the prostate for the treatment of BPO). Therefore, the following studies of comparative data are displayed to demonstrate the inconclusiveness of the existing data sets.

## 3. Body of Evidence: Landmark Studies of HoLEP, ThuLEP, ThuVEP, Bipolar EEP, and Green LEP

Zhang et al. (2012) [34] compared the clinical outcomes between ThuLEP (70 W) and HoLEP (90 W) in a prospective randomized trial with 133 patients and a follow-up (FU)-period of 18 months. ThuLEP required a longer operation time (72.4 vs. 61.5 min), but resulted in less blood loss compared to HoLEP (130.0 vs. 166.6 mL). Catheterization time was comparable. 

At the end of FU, International Prostate Symptom Score (IPSS)decreased by 5.2 in the ThuLEP group and 6.2 in the HoLEP group. Quality of Life (QoL)-score and maximum flow rate (Qmax) were similar between the two groups (1.3 vs. 1.2 and 23.4 vs. 24.2 mL/s). Postvoid residual (PVR) decreased by 82.50% and 81.73% in the ThuLEP and HoLEP groups, respectively. The mean PSA reduction after HoLEP and ThuLEP was 30.43% and 43.36%, respectively. No urethral or bladder neck strictures were found in either group. Obviously, both methods relieved LUTS equally with high efficacy and safety. ThuLEP was statistically superior to HoLEP regarding blood loss, and inferior to HoLEP regarding operation time, although the differences were clinically negligible.

In the same line, Xiao KW et al. (2019) [35] published a systematic review and meta-analysis, including five independent studies and a total of 1010 patients, comparing ThuLEP vs. HoLEP. He noted any statistically significant differences between the groups concerning operation time, enucleation time, morcellation time, catheterization time, and hospital stay. All of the included studies recorded a smaller hemoglobin drop in the ThuLEP-arm; however, this difference was not statistically significant. 

The high enucleation efficacy of the ThuLEP technique was reported in three studies. This could be due to the depth of the wavelength of the thulium laser, which is close to the water absorption peak. Water is the main absorbing substance, which comprises about two-thirds of the prostate; thus, resulting in a high energy absorption rate. Therefore, compared to the pulsed mode of the Holmium:YAG laser, the continuous wave mode of the Thulium:YAG laser might provide faster enucleation. The difference in coagulation depth between the Thulium:YAG laser (2 mm) and the Holmium:YAG laser (4 mm) may be explained; for the final coagulation, a longer time may be required after ThuLEP, compared to HoLEP. Both procedures provide satisfactory micturition improvement of LUTS symptoms, while they appear to have similar adverse event profiles.

Bipolar enucleation of the prostate (BEEP) includes a large range of procedures, such as plasmakinetic enucleation of the prostate (PkEP), transurethral resection enucleation of the prostate (TUERP), bipolar plasma enucleation of the prostate (BPEP), transurethral vapor-enucleation resection of the prostate (TVERP), transurethral vapor-enucleation of the prostate (TVEP) and, finally, bipolar enucleation of the prostate (BipoLEP). Chunxiao Liu et al. (2010) [36] published the effectiveness of the BEEP using the Plasmakinetic™ system, presenting in a series of 1.100 patients with a median follow up of ca. 4.5 years, an indubitable Qmax elevation by 250% (21.7 ± 7.4 mL, 7/s at 6 years postop), PVR reduction by 90.8% (13.1 ± 6.4 mL), IPSS reduction by 79.3% (4.9 ± 1.6 points) and finally, QOL-score reduction by 67.4% (1.5 ± 0.3 points). The PSA-reduction, 6 months postoperatively, showed a decrease of 88.6%. Within the 6-year follow up, postoperative complications included meatal stenosis in nine cases, incontinence in 56, urethral stricture in 12, and bladder neck contracture in only 10 patients. Along the same lines, Lingfeng Zhu et al. (2012) [37], comparing bipolar EEP vs. TURP in an randomised clinical trial (RCT) with 80 patients overall, demonstrated that bipolar EEP achieved greater resected prostate tissue, less blood, shorter catheterization time, and postoperative hospital stay, in favor of EEP. The postoperative improvement in IPSS, QoL, Qmax, and PVR was similar in both groups at 1, 6, 12, and 24 months, but significantly in favor of bipolar EEP at 36, 48, and 60 months. 

Recently, Arcaniolo et al. from the EAU Section of Uro-Technology (ESUT)-Research Group (2019) [33] carried out a meta-analysis that included 14 comparative studies (5 RCTs, 2 cohort prospective non-randomized, 1 propensity score-matched paired analysis, and 6 cohort retrospective studies), with 2317 subjects overall (1178 patients for BEEP and 1139 for bipolareTURP). They demonstrated the clear superiority of BEEP in functional as well as in perioperative outcomes. There was no difference in terms of operative time, but there was a higher amount of resected tissue, and a shorter bladder irrigation and catheter time for BEEP. Lower hemoglobin drop was found in BEEP, while postoperative (short and long-term) complications and transfusion rates were lower for BEEP. The long-term incontinence rate was not statistically different between the two groups. Furthermore, the BEEP group had smaller residual prostate volume and postoperative PSA value, translating into a more “complete resection” compared to b-TURP. There was no difference among the two procedures regarding urethral stricture rate, whereas the re-intervention rate was higher in the b-TURP group.

Thulium-Laser is used in the scenario of vapo-enucleation (ThuVEP), thereby focusing of the vaporizing features of the energy source, as first published by Bach T et al. (2009) [3], or as one energy source option in the context of anatomical blunt dissection (ThuLEP), as described by Herrmann TRW et al. (2010) [4]. In ThuVEP, vapo-incisions into the prostate are carried out in order to resect the sample containing the transitional zone by aiming at the level of the surgical capsule, thereby emulating the template of conventional HoLEP. 

Iacono et al. (2012) [38] demonstrated functional outcomes of ThuLEP (120 W/40 W) in 148 patients in a retrospective study with FU at 12 months. All functional parameters improved: Qmax (8.23 mL/s to 28.67 mL/s), IPSS (21.10 to 3.9 points), QoL (4.38 to 0.94 points), PVR (146 mL to 12.89 mL), prostate volume (108 mL to 13.76 mL), and PSA (9.53 ng/mL to 0.93 ng/mL). Only 2.7% of the patients needed early postoperative blood transfusions due to persistent hematuria with continuous bladder irrigation and prolonged catheterization. Moreover, 6.7% of patients had postoperative irritative symptoms with a temporary urge incontinence and urinary tract infections (UTI) occurring in 12.8% of patients. Only two patients developed urethral strictures during follow-up, which were treated by cold incision. In a structured review, Kyriazis et al. (2015) [29] reported a mean operative time, including morcellation between 70 and 102 min. Blood loss, documented by hemoglobin decrease, was minimal, ranging between 0.5 and 1.27 g/dL. Catheterization time ranged between 2.1 and 2.4 days. PSA reduction, as an indicator of efficiency in adenoma resection, varied between 30.4% and 90%. A clear amelioration of IPSS, Qmax, QoL, and PVR was also described.

Gross AJ et al. (2012) [39], carried out a prospective study with 1080 patients undergoing ThuVEP, classifying the patients according to their prostate volume (<40 mL (median 30 mL)/40–79 mL (median 54 mL)/>80 mL (median 100 mL)). They managed to show an improvement of all functional outcomes in each subgroup with a 45%, 58%, and 63% tissue removal, respectively. The most frequent early complications were urine retention (9%), re-intervention (re-morcellation, secondary apical resection, and clot evacuation) in 4.7% of patients, and hemorrhage requiring blood transfusion in 1.7%. The complications were prostate size-dependent and decreased significantly over time, reflecting the learning curve the procedure. Netsch C et al. (2013) [40] published the long-term results from a prospective analysis of 124 patients who underwent ThuVEP. At the 12-month follow-up, IPSS, QoL, Qmax, and PVR improved significantly compared with preoperative assessment, and continued to do so during follow-up. PSA decreased from 4.7 to 0.92 ng/ml corresponding to a PSA reduction of 83.6% at 12-month follow-up. At 4 years postoperatively, Qmax (20 vs. 7.6 mL/s), PVR (25 vs. 107.5 mL), IPSS (4 vs. 21), and QoL (1 vs. 5) differed significantly from baseline. Major adverse events requiring re-interventions within four weeks after surgery were necessary in 8.1% of the patients. About 6% had postoperative irritative symptoms, which required anticholinergic therapy with a median duration of 30 days. Moreover, 3.2% had postoperative mild urinary stress incontinence, which resolved in all, within median 24.5 (15.8–97) days. Urinary tract infections occurred in 5.6% of patients and urethral stricture and bladder neck contracture developed in 1% and 1.6% of the patients, respectively. Zhu et al. (2014) [41] in a systematic review and meta-analysis compared ThuVEP/ThuLEP (TmLRP) with TURP and analyzed seven studies (four RCTs and three non-RCTs). One year postoperatively, the improvement of Qmax and IPSS favored ThuVEP/ThuLEP while PVR and QoL were not significantly different, although the improvement was greater in the TmLRP group. The operation time of TmLRP was significantly longer than that of TURP, which seemed to be associated with the learning curve. However, in patients with large prostates, TmLRP had an advantage in the operation duration over TURP. Furthermore, TmLRP offered the advantages over TURP in terms of decreased serum sodium, catheterization time, and hospital stay during the perioperative period.

Greenlight consists of kalium-titanyl-phosphate (KTP) or lithium triborate (LBO) with a wavelength of 532 nm and penetration depth of 0.8 to 3 mm. It is absorbed mainly by hemoglobin, which explains its perfect hemostatic action and wide use for BPH treatment in patients on anticoagulants. Firstly, a generator of 80 W (KTP) was developed, followed by a 120 W-generator in 2006 and, finally, a 180 W-generator in 2010. Transurethral anatomical enucleation of the prostate with Greenlight laser was firstly described by Gomez Sancha et al. in 2015 [5]. Panthier et al. 2019 [42] published the first short-term perioperative outcomes in 100 patients treated with Greenlight enucleation of the prostate (GreenLEP) underlying a significant improvement of IPSS, QoL, and Qmax at 1, 6, and 12 months postoperatively, similar to the previous big HoLEP series and conventional TURP. At the end of the follow up period, PVR was reduced, compared to preoperative characteristics (318 ± 267 mL to 95 ± 62 mL), while PSA decreased from a mean of 6.17 ng/mL to 1.06 ng/mL, and prostate volume from 85.8 mL to 24 mL, respectively. Regarding perioperative data, they reported an operating time of 85 min and an enucleation time from 60 min. Capsular perforation was found in 28% of the patients, while re-intervention and conversion to TURP was performed in 2% of the patients. The mean prostatic adenoma removal ran into a mean of 45 g. Six patients reported macrohematuria postoperatively (only one had to be re-operated with coagulation and clot removal), whereas 7 patients suffered urinary tract infections and 16 patients experienced temporary irritative or stress incontinence one month after the procedure. 

Ruszat R et al. [43] analyzed perioperative and postoperative results in 500 patients treated by 80 W-Greenlight-Vaporization. The group noted an overall improvement of Qmax by 108% at the end of a 5 y-follow-up period. IPSS and QoL improved by 58% and 65%, respectively, whereas PSA declined about 50%. Intraoperative bleeding occurred in 3.6%. In terms of early postoperative complications (<30 days), 15% of patients (especially patients with prostate volume >80 mL) suffered from dysuria and 9.8% and 6.8% reported macrohematuria. In regards to long term functional outcomes, retreatment was necessary in about 7%, bladder neck stricture was observed in 3.6%, and urethral stricture in 4.4% of the patients, respectively. Brunken et al. [44] performed a review, which included 10 studies, to prove the efficacy of the 180 W-generator in 1640 patients in 2015. Operation time varied between 40 and 60 min, and laser time varied between 22 and 45 min. All of the examined functional outcomes were found to be improved in contrast to the baseline characteristics in each study. Prostate volume was reduced by 24–61% and was found to be gland size dependent, while PSA was reduced by 37–79%.

Xiao KW et al. (2020) [45] conducted a meta-analysis, including 4 RCTs, to compare the safety and efficacy of diode laser enucleation of the prostate (DiLEP) versus bipolar plasma kinetic enucleation of the prostate (PKEP). The authors concluded that both methods were safe and efficient for the treatment of BPH. However, DiLEP showed less perioperative hemoglobin drop, less postoperative catheterization time, less postoperative irrigation time, and lower rates of postoperative irritative symptoms compared to the PKEP group.

He G et al. (2019) [46], in a retrospective trial with 126 patients randomized in two groups according their transurethral endoscopically enucleation of prostate (DiLEP vs HoLEP), because of BPH, showed the effectiveness and safety of both methods, in which appeared similar perioperative outcomes (operative time, resected tissue, catheterization, and hospitalization length of stay). Furthermore, early or late complications were similar for both groups. Functional outcomes, such as Qmax, PVR, IPSS, and QoL were impressively improved compared to the baseline characteristics; however, were without significant difference between the two groups in the 3-, 6-, or 12-month follow-up. The only parameters in which the DiLEP group showed superiority was less blood loss and the decrease in hemoglobin compared to the HoLEP group.

## 4. Treatment of Patients Under Antithrombotic and Antiplatelet Medication

Rai P et al. (2019) [47], comparing bipolar resection of the prostate against bipolar enucleation (BPEP) in patients under anticoagulation (AC) or antiplatelet (AP) therapy with BPH and prostate volume >60 mL, showed that both methods are safe and effective, but BPEP showed superiority in terms of lower clot retention rate, less irrigation time, and decreased hospital stay. 

El Tayeb MM et al. (2016) [48], in a retrospective study comparing 116 patients on AC/AP therapy with 1558 patients who were free from AC/AP therapy (underwent HoLEP), did not show any significant difference in both groups regarding postoperative transfusion rates and hemoglobin drop. Two patients (1.9%) in the AC/AP cohort required clot evacuation vs. 10 patients (0.7%) in the no AC/AP cohort, while postoperative outcomes were comparable in all aspects, except for length of hospital stay and duration of continuous bladder irrigation, both of which were longer in the AC/AP group. Bishop CV et al. (2013) [49] came to the same conclusion in a retrospective review, in which 125 patients underwent HoLEP (52 patients on antithrombotic therapy at the time of surgery and 73 patients who were not on antithrombotic therapy during surgery). Tyson MD et al. (2009) [50] drew similar outcomes from the data of 76 HoLEP patients (39 patients on AP/AC and 37 controls); in both groups, no patients required any necessary blood transfusions, and there were similar intraoperative bleeding rates. Along the same lines, Yuk HD et al. (2020) [51], observed data from 955 patients (74% free from medication and 26% under AC/AP therapy) who underwent 3-lobe HoLEP, and found similar intraoperative outcomes regarding hemoglobin drop, as well as similar postoperative outcomes regarding bladder irrigation, length of catheterization, and length of hospitalization. Moreover, the Clavien-Dindo Grade >2 complications, at 2 weeks, and 3 and 6 months postoperatively, did not appear any significantly difference. Zheng X et al. (2019) [52] performed a meta-analysis, that included 33 publications, of patients under AC/AP therapy, who underwent HoLEP, and confirmed that patients on antithrombotic/antiplatelet therapy, and those without, have similar hemoglobin decrease during HoLEP, as well as similar intraoperative and postoperative functional outcomes. However, continuous intake of antithrombotic drugs during HoLEP increased the risk of postoperative bleeding, as well as the blood transfusion rates, either in the anticoagulation or in antiplatelet subgroups. Even in this case, the blood transfusion rate (4.0%) was still lower than in TURP (6.4%) and in OP (14%). 

Netsch C et al. (2014) [53] described the ThuVEP procedure in 56 patients under continuous AC/AP therapy (aspirin or clopidogrel or aspirin/clopidogrel, coumarin derivatives) mentioning postoperative clot retention, requiring manual bladder irrigation as well as persistent hematuria and clot retention, requiring transurethral electrocauterization and evacuation of the bladder tamponade in four patients. Moreover, four patients required blood transfusions. After discharge, and in a median of 9.5 days, four patients had to be re-hospitalized for bladder irrigation because of clot retention. Sener TE et al. (2017) [54] recommended ThuVARP in patients on AC/AT therapy, without preoperative bridging with LMWH, in a retrospective study that included 103 patients classified in two groups (group A consisted of 47 patients with low molecular weight heparin (LMWH) bridging preoperatively and group B with 56 patients who were operated on AP/AC therapy). Neither pre- nor postoperative hemoglobin values were statistically significantly different between groups A and B, which approves the safety and effectiveness of the procedure in anticoagulated patients. However, the drop in hemoglobin values in the preoperative and postoperative periods was significantly higher in group A.

Ruszat R et al. (2006) [55] published the perioperative and short-term postoperative results from Greenlight (80 W)-Laser Photo-Vaporization of the Prostate (PVP) of prostate in 116 men under AC/AP therapy (36 receiving coumarin derivatives, 71 aspirin, and 9 clopidogrel) compared to 92 patients without anticoagulant therapy. They observed no bleeding complications necessitating blood transfusions in both groups, while average postoperative decrease of hemoglobin was similar in AC/AP and control group (8.6% vs. 8.8%, respectively). However, postoperative bladder irrigation, due to hematuria, was obligated in almost 17% of the patients under AC/AP therapy, in contrast to only 5% in the control group (p < 0.001). Similar results with 80 W-Greenlight PVP were found by Reich O., Bachmann A et al. (2005) [56] in 66 anticoagulated patients, mentioning neither significant intraoperative bleeding nor postoperative hemoglobin decrease, nor blood transfusion. Meskawi M et al. (2019) [57] controlled the effectiveness and safety from 180 W-Greenlight in a retrospective analysis of 422 patients in four groups (control group, acetylsalicylic acid, antiplatelet agents other than acetylsalicylic acid, and anticoagulation agents). Hematuria grade I rates were higher in group 2 (25%) and group 4 (27%) compared to 9% in group 1 and 8.5% in the control group. No statistically significant difference was recorded for hematuria grade 2. Only one patient on anticoagulation agents required a transurethral electro-coagulation postoperatively. Hemoglobin decrease after surgery was similar among the groups. Two patients with deep baseline hemoglobin required blood transfusion after surgery. Serious bleeding events were present in 16.8%, 16.1%, 16.7%, and 10.8% of patients from groups 1, 2, 3, and 4, respectively. 

Recently, Zheng X et al. (2019) [58] conducted a meta-analysis of 2299 anticoagulated and non-anticoagulated patients in 11 studies undergoing PVP; they published that PVP is not associated with intraoperative bleeding since only one patient (1/2041) in the anticoagulant therapy group (1/616) required blood transfusion. In terms of bleeding, PVP seems to be safe for high-risk patients with BPH, regardless of the administration of anticoagulant therapy. Furthermore, the discontinuation of antithrombotic therapy in patients with comorbidities, particularly cardiovascular disease, requiring continuous anticoagulant therapy, may not be necessary in PVP. 

## 5. Learning Curve

Hwang JC et al. (2010) [59], in a prospective trial of 164 patients, categorized in three groups according to the number of HoLEPs (the first 50 patients, the second 50 patients, and the third 64 patients) proposed at least 50 procedures to manage the significant reduction of intraoperative time and hemoglobin drop, increased resected tissue volume, and reduced intra- and postoperative complications. Kuntz RM et al. (2004) [60] reported that 30 HoLEP-procedures in patients with prostate volume < 50 g were sufficient to achieve professionality. Along the same lines, Du C et al. (2008) [61] demonstrated in a retrospective analysis that the complication rates decreased while experience increased, drawing the line in 13 HoLEP procedures in patients with moderate prostate size.

Regarding the Thulium-laser procedures (ThuVEP/ThuLEP), it was maintained that about 25 procedures might be enough to overcome the learning curve in experienced endourologists. Netsch et al. (2013) [62] presented a prospective study regarding the learning curves of the ThuVEP, with the help of the mentor-based approach, resulting in reasonable enucleation, morcellation, and overall operation efficiency after 8–16 operations, even in cases with unexperienced surgeons.

Chen S. et al. (2014) [63], after evaluating 10 cases that took place under supervision of an endourologist experienced with TURP, concluded that plasmakinetic enucleation of the prostate can be performed with safe, effective, functional, and postoperative results. 

Enikeev et al. (2018) [27] conducted a prospective 1:1:1 trial with 90 patients (30 in each group) in order to evaluate the learning curve between HoLEP vs. Thulium Fiber Laser Enucleation of the Prostate (ThuFLEP) vs. Monopolar Enucleation od the Prostate (MEP); they concluded that EEP can be safely performed after 30 surgeries under the supervision of a specialist. Similarly, Robert G et al. (2015) [64] published a prospective multicenter trial to describe the step-by-step learning curve of HoLEP; the operating time and the enucleation technique (mostly the identification of the correct layer) seemed to be the main problem for beginners. More than half of the surgeons who tried to perform an enucleation without the leadership of a specialist did not manage to finish the training course.

Peyronnet B et al. (2017) [22] compared the learning curves and the perioperative outcomes between GreenLEP and HoLEP, considered the learning curve as something to be overcome by managing a trifecta (complete enucleation and morcellation within <90 min and without any conversion to standard TURP) or a pentafecta (trifecta without postoperative complications or stress urinary incontinence at 3 months postoperatively). Operative time and operative time/prostate volume decreased during the first procedures, plateauing from the 30th cases for both GreenLEP and HoLEP, respectively. Trifecta was achieved in four consecutive patients after the 14th case in the GreenLEP group and after the 22nd case in the HoLEP group, whereas Pentafecta was achieved in four consecutive patients after the 18th procedure in the GreenLEP group and after the 40th procedure in the HoLEP group. 

## 6. Elderly Patients

The actual literature regarding the use of lasers in elderly patients is very limited. Piao et al. (2016) [65] controlled the effect of HoLEP in patients classified according to their age (50–59, 60–69, 70–79, and ≥80 years), and maintained that HoLEP was a safe and effective treatment for BPH among the elderly, since postoperative complications were similar in all groups, and in 6-month postoperatives, there were no significant differences in IPSS, quality of life, Qmax, and PVR among the groups (*p* > 0.05). Castellani et al. (2019) [66] maintained the same opinion, observing the effect of ThuLEP in patients under and above 75 years. The functional outcomes, as well as the complications postoperatively, were comparable among the two groups. 

## 7. Incontinence

The incidence of transient early postoperative stress urinary incontinence is, firstly, a function of the learning curve and, secondly, based on the presence of a pre-existing overactive bladder due to benign prostate obstruction. Moreover, the volume of the prostatic gland, the duration of obstruction, and pre-existing neurologic conditions play an important role. 

Houssin V et al. [67] in a retrospective multi-central analysis, involving 2346 patients after HoLEP, came to the conclusion that urinary stress was observed in 14.5% of patients at 3 months, and in 4.2% of patients at 6 months after the procedure. At 3 months postoperatively, increased age, elevated BMI, preoperative urinary drainage, increased enucleated tissue weight, and an experienced surgeon (with at least 40 cases) were significantly associated with urinary incontinence. At 6 months postoperative, increased age, elevated BMI, increased whole gland volume, and diabetes disorder were factors that significantly elevated the incidences of urinary incontinence.

Regarding the continence anatomical structures, taking into consideration that early separation of the apical part of the prostate from the external sphincter can lead to preservation of the sphincter structure, and to reduction of postoperative incontinence rates [68], the overarching principle of all EEP approaches, regardless of the energy power, consist of anatomical preparation, anterior–posterior dissection, early release of the ventral mucosa, and apical mucosa sparing incisions in order to reduce early postoperative incontinence [68].

## 8. BipoLEP

Hirasawa et al. (2018) [69] analyzed, retrospectively, data from 584 patients after undergoing bipolar enucleation of the prostate, and cited postoperative transient urine incontinence in 17.3%, 13.5%, 3.1%, 0.41%, and 0%, at 1, 3, 6, and 12 weeks, respectively, mentioning that age and prostate volume were significant independent risk factors for transient urinary incontinence.

## 9. HoLEP

Postoperative stress urinary incontinence was found in 4.5% patients; however, pelvic floor exercises were associated with recovery of most patients after three months, with persistent incontinence in only 0.5% of the patients (5/978). Necessary anticholinergic medications as a result of urge symptoms were reported in 1.3% of patients [70]. Along the same lines, Elmansy HM et al. in a retrospective analysis of 949 patients, after HoLEP procedure with a FU of 10 years, found persistent urge and stress incontinence in 1% and 0.5% of patients at the end of FU, respectively [71].

## 10. ThuLEP

Kyriazis et al. [29], with a review of literature regarding ThuLEP procedures, reported transient irritative symptoms between 6.7% and 18.5 %, with no patient reporting symptoms at the end of each study period. Urge urinary incontinence was reported in 6.7% of patients in the study by Iacono et al. [39], recruiting patients with large prostate volume, but none of the patients had incontinence at 12 months.

## 11. ThuVEP

Postoperative irritative symptoms consisted of transient urge and stress incontinence (after ThuVEP procedures), and reportedly occurred in 7% and 18% of the patients; however, these adverse events usually resolved spontaneously or with conservative treatments (i.e., anti-inflammatory drugs, antibiotic therapy, and pelvic floor exercise). At 1 year postoperatively, the end of the 12-month follow-up, the incidence of storage symptoms (range 0–4.8 %), urge incontinence (range 0–1.8%), and stress incontinence (range 0–3.6 %) had significantly decreased [72].

## 12. GreenLEP

Panthier F et al. [42] in a retrospective analysis with 100 patients who underwent GreenLEP, and a follow-up at 12 months, mentioned urinary urgency or stress urinary incontinence in 16 patients (16%) one-month post procedurally. At 6 months, only 2% of patients suffered under urinary urgency symptoms, and one patient (1%) had persistent stress incontinence at the end of the follow-up period.

## 13. ThuLEP vs. HoLEP

Xiao K et al. [35], in a systematic review that included 1010 patients from five independent studies comparing ThuLEP vs. HoLEP, did not demonstrate any significant postoperative incontinence rates between the two groups at 1 and 6 months after the operation (OR 0.61 (0.33, 1.13), *p* = 0.12 and OR 0.52 (1.10, 2.86), *p* = 0.45, respectively). However, due to the deeper penetration depth of Holmium-Laser, Thulium laser may result in less irritative symptoms directly after postoperatively.

## 14. HoLEP vs. BipoLEP

Guo et al. [73], comparing HoLEP vs. bipolar plasmakinetic prostatectomy in a review that included seven studies with 2031 patients, failed to show superiority of one method over the other, and referred ending the follow-up time of 12 months.

## 15. GreenLEP vs. HoLEP

Comparing GreenLEP to HoLEP, the rates of stress urinary incontinence between the two groups did not differ significantly at 1 month (26% vs. 24%; *p* = 0.77), 3 months (9% vs. 6%; *p* = 0.42) or 6 months post-surgery (2% vs. 0%; *p* = 0.53) [22].

## 16. ThuVEP vs. HoLEP

At the one-month follow-up, nearly 11% and 4% of patients complained about urge incontinence and stress incontinence, respectively, in the HoLEP group, compared to 5% and 2.5% in the ThuVEP group. Patient continence recovered within 6 months. No patients had persistent irritative urinary symptoms or incontinence in a retrospective analysis of 88 consecutive patients [74].

## 17. Sexual Function—Retrograde Ejaculation

Since the majority of patients with BPO are at an advanced age, with many being under 5α-Reductase inhibitors (5-ARIs) medication, their sexual lives were, before the operative treatment, already impaired. Independent of this fact, a transurethral operative treatment could variously affect the sexual function of patients.

In comparison to transurethral resection of the prostate, EEP appears to have better long-term benefits with regard to sexual function of patients, translating into higher International Index of Erectile Function (IIEF) scores, while they present similar high retrograde ejaculation rates [75].

TURP series reported retrograde ejaculation rates of 50–78% as well as erectile dysfunction rates of 6.5% [76,77]. HoLEP showed comparable retrograde ejaculation rates of 50–76% when compared to TURP [77]. Erectile function showed higher scores in the IIEF-15 questionnaire in the HoLEP group when compared to the control group (patients with LUTS without any operative treatment) in the prospective analysis from Elsah AM [78]. Sexual desire, intercourse satisfaction, and overall satisfaction scores were similar in both groups, while with ejaculatory dysfunction, according to the Male Sexual Health Questionnaire for Ejaculatory Dysfunction (MSHQ-EjD) score, there appeared to be a significant difference between HoLEP and control groups, with decreased ejaculatory volume being the most common ejaculatory abnormality after HoLEP. The same author described, in a previous study, worsened erectile function in 17.2% of the HoLEP group vs. 29.3% of patients after PVP procedure.

Pushkar et al. [79], performing a prospective analysis of 119 patients overall, randomized in two groups according to their treatment (63 patients HoLEP vs. 56 TURP), and concentrating exclusively on their sexual function postoperatively, could not demonstrate any significant difference between the groups with regard to erectile function at the end of the 6-month FU. Moreover, the quality score for orgasm, sexual desire, and intercourse satisfaction score, were significantly better in the HoLEP group compared to the TURP group. Furthermore, age seemed to be a significant factor for the convalescence of sexual function in both groups.

Comparing GreenLEP vs. Greenlight PVP, GreenLEP showed a significant amelioration of EF postoperatively (only 2% of GreenLEP-arm exhibited declined EF vs. 19% of Greenlight PVP) [80]. On the other hand, in the same study, there was only 1.2% of patients with antegrade ejaculation after GreenLEP vs. almost 27% in the PVP group.

ThuLEP showed, in small prospective studies, unimpaired erectile function preoperatively and postoperatively with similar IIEF-scores, with ejaculation conserved in ca. 52% of patients, according to a MSHQ-EjD questionnaire [81,82,83].

Ejaculatory function-sparing (ES) techniques are described in order to prevent the antegrade ejaculation. In this way, antegrade ejaculation was preserved in 72% of patients at three months and 77% at six months after ThuLEP-ES without erectile function, before and after surgery. The reported antegrade ejaculation rates were 90.8% and 46.2% for the ejaculation sparing modifications of TURP, HoLEP, respectively [84,85].

## 18. Discussion

Endoscopic Enucleation of the Prostate (EEP) has been, since 2016, the gold standard in the treatment of BPO in patients with middle and large prostatic glands, replacing, in an effective and safe way, the open adenectomy. The development of laser technology initially led the discourse on lasers and focused on their different properties rather than technique or surgical anatomy, respectively. In and after 2016, the discussion ultimately moved towards surgical technique and accepting anatomical preparation as the common of all EEP techniques (AEEP). A plethora of studies based on EEP, with a variety of different laser-sources inclusive of bipolar technology were undertaken in order to establish the best procedure. At the end of the day, enucleation is enucleation [Herrmann TRW, 2016] [8]. EEP can be safely performed, with all of the available laser sources (even with a diode laser, if used adequately), as well as with bipolar plasmakinetic energy with durable efficacy outcomes (IPSS and Qmax), and safety profiles (transfusion, infection, stricture, etc.) (Gu C et al., 2019) [86]. The body of evidence is limited with regard to direct comparison of the different power sources. However, objective data suggest that the treated volume is the same, while the surrogate parameter is PSA reduction (see Table 1). The functional data seem to be equivalent as well. To date, no superiority has been substantiated with regard to the functional improvement (see Table 2). Although the magnitude of data is on BipoLEP and HoLEP, the given literature of the other treatments in the long-term follow up matches the expectations of EEP. 

With regard to anticoagulation and antithrombotic medication, it must be underlined that the results from the vaporizing approach cannot be directly transferred into the enucleation scenario. However, EEP appears to clearly benefit compared to TURP, which is characterized by higher bleeding/transfusion rates. In this topic, the actual literature consisted of retrospective studies with a small number of patients, with lack of long-term evidence and large heterogeneity. Nevertheless, existing data, again, suggest that bipolar technology, as well as Holmium-/Thulium/Greenlight- and Diode-laser show comparable results concerning blood loss and transfusion rates, offering a certain option within the bounds of anatomical enucleation of prostate. The EAU guidelines recommend laser vaporization and enucleation of the anticoagulated patients, while according to American Urological Association (AUA) guidelines, HoLEP, PVP, and ThuLEP should be considered in patients who are at higher risk of bleeding, such as those on anti-coagulation drugs. 

Postoperative irritative symptoms after a transurethral enucleation of the prostate seems to be more associated with other parameters than energy source. The actual literature failed to show superiority of an energy power over the others in the topic of postoperative incontinence [11]. Therefore, the volume of the prostatic gland and the enucleation method itself, as well as a pre-existing pathology of the bladder storage, or voiding function because of BPO, are important factors, which should be considered before the operative treatment takes place. In all of the cases, the vast majority of transient postoperative urge incontinence and urge symptoms were sufficiently treated with anticholinergic drugs.

With reference to the learning curves, a direct comparison between the powers is, for multiple reasons, difficult. The literature is characterized mostly by subjective position, which is strongly associated with the endourologic experience of the authors. Generally, enucleation can safely be performed after a learning curve of 15–40 procedures in a mentorship situation.

## 19. Conclusions

In the armamentarium of contemporary surgical treatment of benign prostatic obstruction (BPO), transurethral anatomical enucleation of the prostate (AEEP) plays a central role in large prostate glands. The EAU guidelines on management of male lower urinary tract symptoms (LUTS) position both laser and current based techniques as equally valid options in the algorithm of treatment. This was achieved by the academic discourse on the suitability of different energy sources to perform enucleation inspired by the upcoming novel Thulium laser in the mid-2000s, and finally moved to surgical technique and anatomical considerations. Bipolar enucleation has proven to be safe and effective, as well as durable in the long-term, and successful in several other scenarios, such as in patients on anticoagulation and antithrombotic medications, as well as in patients highly concerned with preservation of unhampered sexual functions. Therefore, a laser is a solid versatile option in performing enucleation. Today, no thoroughly convincing data on the superiority of one energy source over the other exists at any end point. Therefore, in the sense of critical importance, AEEP can be safely and effectively performed using any technology without compromising postoperative outcome.

## Figures and Tables

**Table 1:** PSA-Drop.

Author	Technique	PSA-Reduction (%)
Timmouth et al. 2005 [87]	HoLEP	81.7–86
Netsch et al. 2015 [72]	ThuVEP	81–88
Herrmann et al. 2010 [4] Kim et al. 2015 [88]	ThuLEP	83–93
Kim et al. 2008 [16]	TmLRP-TT, Tangerine	82.5
Misrai et al. 2016 [89]	GreenLEP	67
Peyronnet et al. 2017 [22]	HoLEP vs. GreenLEP	83.1 vs. 86.5
Zheng [90]	DiLEP vs. BEEP	91.5 vs. 92.6

**Table 2:** Long-term functional outcomes of BPH treatments. (HoLEP = Holmium Laser Enucleation of the Prostate, ThuVEP = Thulium Vapoenucleation of the prostate, ThuLEP = Thulium Enucleaion of the prostate, TmLPR − TT = Thulium laser resection of the prostate-tangerine technique, DiLEP = Diode Laser Enucleation of the prostate).

Publikation	Study	Patients	Technique	FU (months)	Median Prostate-volume (mL)			IPSS/AUA-SS			QoL			Qmax (mL/s)			PVR (mL)			Urethra stricture	Bladder neck contracture	Recurrent adenoma
					preop	Postop (months)	FU	preop	Postop (months)	FU	preop	Postop (months)	FU	preop	postop	FU	preop	postop (months)	FU			
														
Kuntz et al. 2008	RCT	100	HoLEP	60	114	-	-	22.1	-	3.0	-	-	-	3.8		24.3	280	-	10.6	4	3	1
		100	OP		113	-	-	21.0	-	3.0	-	-	-	3.6		24.4	292	-	5.3	3	3	0
Gilling et al. 2011	RCT		HoLEP	84	77.68	28.4 (6 m)		26.39	6.1 (6 m)	8.0	4.79	1.25 (6 m)	1.47	8.28		22.09	116	33.7 (6 m)	-	-	-	0
			TUR-P		70.0	46.6 (6 m)		23.72	5.2 (6 m)	10.3	4.7	1.25 (6 m)	1.31	8.26		17.83	126	51.8 (6 m)	-	-	-	3
Ahyai et al. 2007	RCT	100	HoLEP	36	53.5	-	-	22.1	1.7 (12 m)	2.7	-	-	-	4.9	27.9 (12 m)	29.0	238	2.3	8.4	4	3	1
		100	TUR-P		49.9	-	-	21.4	3.9 (12 m)	3.3	-	-	-	5.9	29.9 (12 m)	27.5	216	26.6	20.2	3	3	0
Chen et al. 2014	RCT	80	BipoLEP	72	110	-	-	25.6	4 (6 m)	3	4	1 (6 m)	2	4	24.3 (6 m)	25.4	240	14.5 (6 m)	20	3	1	0
		80	OP		114.5	-	-	25.7	4 (6 m)	3	5	1 (6 m)	2	4	24.0 (6 m)	25.9	249	14.5 (6 m)	16.5	1	4	0
Zhu et al. 2013	RCT	40	BipoLEP																			
		40	TUR-P																			
Yang et al. 2016	RCT	79	ThuLEP	60	72.4	-	-	22.7	5.2 (12 m)	6.5	-	1.2 (12 m)	1.4	8.7	23.2 (12 m)	19.4	79.5	27.4 (12 m)	34.9	0	0	-
		79	TUR-P		69.2	-	-	23.4	4.6 (12 m)	6.9	-	1.1 (12 m)	1.5	9.1	23.9 (12 m)	18.9	72.4	28.3 (12 m)	32.6	0	0	-
Netsch et al. 2016	prospective	500	ThuVEP	72	50	13 (12 m)	-	21	3.5 (12 m)	5	4	1 (12 m)	1	6.9	21.8 (12 m)	16.3	130	25 (12 m)	35.9	4	4	3
Riecken et al. 2014	prospective	269	Greenlight (80 W)	60	52.3	-	-	19.4	-	12.8	3.7	-	2.4	8.3		6.8	119.5	-	85	7	3	10
		127	TUR-P	60	44.2	-	-	18.4	-	13.5	3.7	-	2.8	10.0		12.6	95.6	-	64	1	0	2
Bachmann et al. 2015	prospective	139	Greenlight (180 W)	24	48.6 (12 m)	21.9	23.9	21.2	6.9 (12 m)	6.9	4.6	1.4 (12 m)	1.2	9.5	22.9 (12 m)	21.6	110.1	42.8 (12 m)	45.6	3	8	3
		142	TUR-P	24	46.2 (12 m)	21.0	22.4	21.7	5.7 (12 m)	5.9	4.5	1.5 (12 m)	1.2	9.9	24.7 (12 m)	22.9	109.8	33.4 (12 m)	34.9	5	3	1
